# Perception towards Online Teaching-learning in Medical Education among Medical Students during COVID-19 Outbreak in Nepal: A Descriptive Cross-sectional Study

**DOI:** 10.31729/jnma.5410

**Published:** 2021-02-28

**Authors:** Rakesh Singh, Madhusudan Subedi, Smriti Pant, Pragya Rai, Krishna Kumar Gupta, Ambika Thapa Pachya, Kaushal Kumar Singh, Abdul Sami Khan, Kishor Adhikari, Shanta Sharma, Sanjeev Shah, Babita Singh

**Affiliations:** 1 Patan Academy of Health Sciences, Lalitpur, Nepal; 2 National Medical College Teaching Hospital, Birgunj, Nepal; 3 Kathmandu University School of Medical Sciences, Dhulikhel, Nepal; 4 Chitwan Medical College Teaching Hospital, Bharatpur, Nepal; 5 Devdaha Medical College and Research Institute Pvt. Ltd, Rupandehi, Nepal; 6 Universal College of Medical Sciences, Bhairahawa, Nepal

**Keywords:** *medical education*, *medical students*, *online teaching-learning*, *perception*

## Abstract

**Introduction:**

The outbreak of coronavirus disease in Nepal led medical colleges to suspend inperson teaching-learning activities and ultimately online platform was introduced to deliver the contents of medical education. The objective of this study was to describe the perception of medical students towards online teaching-learning introduced during the COVID-19 outbreak in Nepal.

**Methods:**

An online survey using a descriptive cross-sectional study design was carried out among 515 undergraduate medical students currently enrolled in medical colleges in Nepal. A semi-structured questionnaire in Google form was utilized to collect data. The link of the Google form was sent to the potential respondents through email and social media. Descriptive statistics, including frequency, percentage, mean, and standard deviation were used to analyze data in SPSS vs20. Ethical approval was sought from Nepal Health Research Council to conduct this study, and digital informed consent was taken from study respondents.

**Results:**

The overall score of perception of online teaching-learning was 17.61±7.19, which indicated many problems in this method of teaching-learning. The mean score of perception of online teaching-learning was found to be different across sex, location of enrolled medical colleges, having a personal electronic device, having an internet connection at residence, having separate room/space for attending online classes, and self-rated computer skills. Moreover, only 28 (5.4%) of respondents had perceived online teaching-learning as a better method of delivering content of medical curricula.

**Conclusions:**

Surveyed medical students in Nepal were found to perceive many problems in online teaching-learning. Moreover, management and faculty members need to take the necessary measures for enhancing the online teaching-learning quality.

## INTRODUCTION

With coronavirus disease (COVID-19) hitting Nepal, the Government enforced lockdown and travel restrictions,^1^ which led medical colleges to suspend inperson academic activities of undergraduate medical students. This suspension would lead to a loss of learning time, lower students' self-esteem, confidence, and loss of clinical skills.^2^ To reduce this loss, medical colleges in Nepal felt the need to use information technology to deliver medical curricula,^3-4^ and resumed undergraduate medical education through online teaching-learning platforms.

The success of online learning hinges on accessibility, usage of appropriate methods, and course content.^5,6^ Online teaching provides both opportunities and challenges to students.^7,8^ The use of an online platform to deliver medical education is comparatively new in Nepal and its effectiveness from students' perspective is a must.

Hence, this study aimed to describe medical students' perception towards online teaching-learning introduced during the COVID-19 outbreak in Nepal.

## METHODS

A web-based cross-sectional descriptive study was carried out among the Bachelor of Medicine and Bachelor of Surgery (MBBS) students currently enrolled from the first year to final year at medical colleges of different provinces in Nepal, who were 18 years or above, and who gave digital informed consent to participate. The data was collected via a web-based online survey by sending the link of the semi-structured questionnaire in the form of a Google document to the undergraduate medical students enrolled in medical colleges in Nepal by email and/or social media via purposive sampling. Medical students from 12 different medical colleges were approached for their participation in the survey. These 12 colleges were selected purposively in a way that we covered medical colleges from all provinces where medical colleges are located. The minimum sample size was estimated using the following formula:


$$\begin{array}{ll} \text{n} & ={\text{Z}}^{\text{2}}\times \text{p}\times \text{q}/{\text{e}}^{\text{2}}\\ & ={\left(1.96\right)}^{2}\times (0.5)\times (1-0.5)/{\left(0.05\right)}^{\text{2}}\\ & =384 \end{array}$$

Where,

Z = 1.96 for confidence interval at 95%
p = prevalence 50%
q = 1-p
e = margin of error 5%

With a non-response of 20%, the estimated minimum sample size was 461. However, with a larger response than expected, the total number of participants enrolled in the study was 515 within a span of one week of the online survey (12 July to 18 July 2020). Ethical approval was taken from the Ethical Review Board of Nepal Health Research Council (NHRC) to conduct the study (reference number 2810).

The questionnaire used in the online survey assessed background characteristics and perception towards online teaching-learning. The part of the questionnaire assessing perception towards online teaching-learning comprised of: a rating scale adapted from the subscale of students' perception of learning of Dundee Ready Educational Environment Measure,^9-11^ and questions exploring perceived experiences of online teaching-learning which were adapted from data collection tool from a similar study.^12^ The adapted rating scale consists of 12 items with five points Likert scale (0 to 4). Each of the twelve items with a mean score of ≥3.5 is true positive points; ≤2 are problem areas; between ≥3.5 and ≤2 indicate aspects that could be enhanced.^9-11^ The maximum score from the rating scale that an individual can obtain is 48 and are interpreted as follows: 0-12 (≤25% score)= very poor, 13-24 (25-50% score)= many problems, 25-36 (50-75% score)= more positive than negative, 37-48 (≥75% score)= excellent.^9-11^ Moreover, the adapted questions assessing perceived experiences of online teaching-learning explored the preference of online teaching-learning method over in-person, attendant of the online class, enjoyment during online class and its reason, duration of active enjoyment during online class, perception of optimal mix of the online and in-person class, and recommended duration of online class per day.

Data collected via the online survey were imported to an excel sheet, cleaned, and analyzed in SPSS (Statistical Package for Social Sciences) version 20.

## RESULTS

The age of the respondents ranged from 18 to 29 years, with a mean age of 21.09±1.96 years. Out of the total 515 respondents, 284 (55.1%) were female; 146 (28.3% ) were enrolled in the first year; 324 (62.9%) were enrolled in medical colleges at Bagmati Province. While most of them 488 (94.8%) had their personal electronic device for attending online classes, a quarter 128 (24.9%) did not have a separate internet connection at their residence, and around one-third 162 (31.5%) did not have a separate room/space where they could attend online classes. Regarding self-rated computer skills, 216 (41.9%) responded as having only basic skills.

Individual item-wise analysis of the rating scale, none of the items on respondents' perception of online teaching-learning scored more than 3.5 (Table 1). Furthermore, the overall score of respondents' perceptions of online teaching-learning was 17.61±7.19 (36.69% of maximum score) (Table 2).

**Table 1:** Mean score of individual items of rating scale of students' perception of online teaching-learning (n = 515).

SN	Scale items	Mean ± SD
1	I am encouraged to participate in online class	1.29 ± 1.01
2	The online teaching is often stimulating	1.14 ± 1.08
3	The online teaching is student centered	1.07 ± 1.01
4	The online teaching helps to develop my competence	1.09 ± 1.05
5	The online teaching is well focused	1.02 ± 1.02
6	The online teaching helps to develop my confidence	1.71 ± 1.19
7	The online teaching time is put to good use	1.47 ± 1.03
8	The online teaching over-emphasizes factual learning	2.53 ± 1.10
9	I am clear about the learning objectives of the course delivered in online class	1.31± 1.13
10	The online teaching encourages me to be an active learner	1.31 ± 1.15
11	Long term learning is emphasized over short term learning in online class	1.99 ± 1.24
12	The online teaching is too teacher-centered	1.67 ±1.26

**Table 2:** Score of overall students' perception of online learning (n = 515).

Characteristics	No. of items	Obtainable score	Mean	SD	Mean Percent
Students' perception of online learning	12	0-48	17.61	± 7.19	36.69

There was a difference in the score of respondents' perceptions of online learning across sex, provincial location of enrolled medical colleges, having a personal electronic device, having an internet connection at residence, having separate room/space for attending online classes, and self-rated computer skills (Table 3).

**Table 3:** Students' perception of online teaching-learning score across selected background characteristics (n = 515).

Characteristics	Categories of characteristics	Frequency n (%)	Students' perception of online learning mean score (SD)
Sex	Male	231 (44.9)	16.85 ± 7.12
	Female	284 (55.1)	18.22 ± 7.21
Year of enrolment	First-year	146 (28.3)	17.62 ± 6.48
	Second-year	118 (22.9)	17.00 ± 6.77
	Third-year	133 (25.8)	17.80 ± 7.59
	Fourth/Final year	118 (22.9)	17.97 ± 8.01
Location of enrolled medical college (Province-wise)	Province 1	42 (8.2)	16.38 ± 6.27
	Province 2	46 (8.9)	14.80 ± 6.82
	Bagmati Province	324 (62.9)	17.94 ± 6.95
	Gandaki Province	65 (12.6)	19.78 ± 8.02
	Province 5	38 (7.4)	15.76 ± 7.89
Have personal electronic device for attending online classes	Yes	488 (94.8)	17.78 ± 7.12
	No	27 (5.2)	14.44 ± 7.91
Internet connection at residence	Yes	387 (75.1)	18.36 ± 7.14
	No	128 (24.9)	15.33 ± 6.89
Separate room/space at residence for attending online class	Yes	353 (68.5)	18.62 ± 7.17
	No	162 (31.5)	15.40 ± 6.76
Self-rated computer skills	Basic	216 (41.9)	16.65 ± 7.38
	Intermediate	268 (52.0)	18.07 ± 6.87
	Advance	31 (6.0)	20.23 ± 7.79

Only 5.4% of the respondents perceived online teaching-learning as a better method in medical education (Table 4). Regarding active engagement in an online class of two hours, 39.7% of the respondents were active for less than half an hour, and none were active for the whole two hours of the session. Three hundred forty six (73.5%) of the respondents did not enjoy online learning sessions because of the major reason for teaching and lack of sufficient discussion 174 (50.3%). Further, when asked how many hours of online class per day they would recommend for delivering the medical curriculum content, the respondents' answers ranged from 0 to 4 hours with a mean of 2.24±0.78 hours.

**Table 4:** Perceived experiences of online teaching-learning and its reasons.

Characteristics	Category	n	(%)
Attended online class, n=515	Yes	471	(91.5)
	No	44	(8.5)
Duration of active engagement in an online class of 2 hours, n=471	Half an hour (30 minutes or less)	187	(39.7)
	An hour	206	(43.7)
	One and half hours	78	(16.6)
Enjoyed online classes and are helpful for learning, n=471	Yes	125	(26.5)
	No	346	(73.5)
Reason for not enjoying online classes, n=346	Class was mostly one-way and did not have enough discussion	174	(50.3)
	Sessions were too difficult to understand	66	(19.1)
	Felt lonely attending online class	38	(11)
	Did not learn anything from online class	15	(4.3)
	Class schedule was inconvenient	12	(3.5)
	Others (inadequate preparation by facilitators, unorganized content, lack clinical and practical skill session and repetitive content)	41	(11.8)
Preferred online teaching-learning over in-person method, n=515	Yes	28	(5.4)
	No	487	(94.6)

## DISCUSSION

The overall mean score of students' perceptions towards online teaching-learning in the current study was 17.61, indicating that there were many problems in the online teaching-learning method. The finding is supported by a study conducted in Pakistan where most medical students had a negative perception of online learning.^3^ One of the reasons for this negative perception and ineffectiveness in online teaching-learning was the lack of skill-building and laboratory sessions in this method of delivering medical education. This is consistent with medical students' attitudes in studies in various nations, including Pakistan, China, Malaysia, and Singapore.^3,13-15^ Moreover, differences in the score of perception of online teaching-learning were found across characteristics of students, including computer skills, availability of the personal electronic device, and internet connection at residence. This is logical as online teaching-learning requires reliable internet connection and sound skills on software and hardware.^16^ However, there was a similar score of perception of online teaching-learning among respondents of different enrollment years. This may be because whatsoever be the year of enrolment, the medical students in Nepal are on the same page regarding exposure to online teaching-learning, as it is a relatively new pedagogy method adopted in medical education in Nepal.

The mean score of the item regarding getting encouraged to participate in online classes in this study was 1.29, indicating a problem area that needs to be emphasized while delivering medical education contents via the online method. The lack of effective participation in an online class was found among students in the current study, with 61.3% feeling lonely due to inadequate discussion during online sessions. This result is congruent to the study done in Pakistani medical students, where 84% of students felt increased isolation due to low student-teacher interaction during online teaching-learning sessions.^3,17^ Similar responses were observed in a study conducted among Polish medical students where the majority reported to be less active during online learning.^18^ The reason for this inactivity may be a lack of an interactive approach while preparing and conducting online medical education sessions.^18^ Similarly, only 26.5% of the present study students perceived online teaching-learning classes to be positive and helpful. The Pakistani study showed similar results where 14% of the medical students considered online learning impact to be positive.^3^

Furthermore, the problem area in online learning indicated by the score of perception of online teaching-learning was also evident from the proportion of students (only 5.4%) who perceived online teaching-learning as a better method of learning in medical education. This finding is similar to the study conducted in Pakistan, where 85% of the medical students preferred traditional in-person teaching over online teaching.^3^ This finding is also consistent with other studies where a majority of the students preferred face-to-face learning.^19-20^ However, studies conducted in Korea and Oman suggested that medical and nursing students preferred and were satisfied with e-learning and digital lectures.^21-22^ The reason for this discrepancy may be that online learning introduced during the lockdown phase in Nepal is new for medical education in Nepal as compared to medical education in Korea and Oman. Furthermore, lack of interaction with patients in the clinical setting and lack of clinical skill learning sessions may be the reasons for fewer adherences to a perceived preference towards online teaching-learning among medical students.

To some extent, use of virtual laboratory materials and virtual patients can be utilized to train medical students and prepare them before encountering a real-life patient,^18,23^ and this, in turn, may help generate enthusiasm in online learning among the students during the crisis situations like COVID-19 outbreak where the in-person teaching-learning activity is a challenge. However, as depicted from the current study's findings, medical colleges' management and faculty members need to frame online classes of not more than two hours session a day. Nevertheless, each online session should not be more than an hour based on an active engagement during an online class.

Moreover, the negative perception towards online teaching-learning in the present study is also reflected by the proportion of the respondents (73.5%) who did not enjoy online classes. This finding is in contrast with the study done in the United States, where 59% of the medical students enjoyed online learning.^12^ This discrepancy in the level of enjoyment of online learning may be due to prior exposure to virtual learning in medical schools in high-income countries.

The limitations of the study include the design of the study because of which the findings need to be interpreted with caution as it does not establish causation. Furthermore, due to the nature of the study being a web-based online survey, potential respondents who were not in access to the internet during the data collection phase might have been left out whose perspective would have been important for the study.

## CONCLUSIONS

Surveyed medical students in Nepal perceived many problems in online teaching-learning in medical education. The majority of the medical students did not perceive online teaching-learning as a better method of online teaching-learning. In this regard, medical educators in Nepal should emphasize addressing the problem areas in online teaching-learning methods, thereby enhancing medical education quality in an interrupted way during emergencies. Further study is recommended to determine the perception of teaching medical education via online platforms among faculties as teachers' perception and experience of delivering education contents via virtual platform is an important aspect to be explored to enhance the quality of teaching-learning methodology medical education.
